# Supplementary material for: Isolation and Characterization of Two Novel Lytic Bacteriophages against Salmonella typhimurium and Their Biocontrol Potential in Food Products
Source: Foods. 2024 Sep 28;13(19):3103. doi: 10.3390/foods13193103 (PMC11476292; doi:10.3390/foods13193103)
Supplement: Supplementary file 1 [file foods-13-03103-s001.zip › foods-3207194-supplementary.pdf]

Table S1. The chloroform sensitivity of phage SPYS_1 and SPYS_2

Salmonella phage	Replicates	The control group (PFU/mL)	Chloroform treatment (PFU/mL)
SPYS_1	1	4.30E+10	4.20E+10
SPYS_1	2	7.10E+10	1.04E+11
SPYS_1	3	9.20E+10	7.80E+10
SPYS_2	1	2.00E+09	1.50E+09
SPYS_2	2	2.00E+09	2.10E+09
SPYS_2	3	2.00E+09	2.00E+09

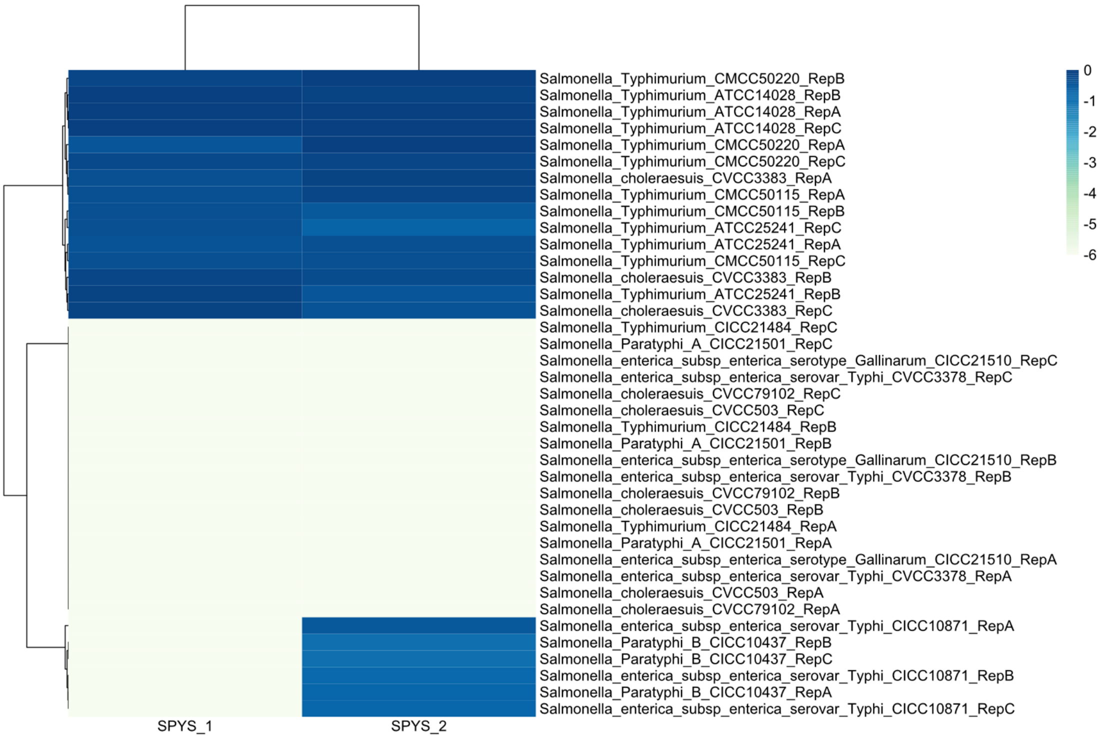

Figure S1. Efficiencies of plaquing results of two *Salmonella* phages against standard strains of *Salmonella* with different stereotypes. The EOP for each phage on each strain represented the relative value of the titer compared to the highest titer of that specific phage across all strains.
